# Deletion of CD38 and supplementation of NAD^+^ attenuate axon degeneration in a mouse facial nerve axotomy model

**DOI:** 10.1038/s41598-020-73984-3

**Published:** 2020-10-20

**Authors:** Yuji Takaso, Masao Noda, Tsuyoshi Hattori, Jureepon Roboon, Miyako Hatano, Hisashi Sugimoto, Charles Brenner, Yasuhiko Yamamoto, Hiroshi Okamoto, Haruhiro Higashida, Makoto Ito, Tomokazu Yoshizaki, Osamu Hori

**Affiliations:** 1 Department of Otolaryngology-Head and Neck Surgery, Graduate School of Medical Sciences, Kanazawa University, Kanazawa, Japan; 2 Department of Neuroanatomy, Graduate School of Medical Sciences, Kanazawa University, 13-1 Takara-machi, Kanazawa, Ishikawa 920-8640 Japan; 3 Department of Biochemistry, Carver College of Medicine, University of Iowa, Iowa City, USA; 4 Department of Biochemistry and Molecular Vascular Biology, Graduate School of Medical Sciences, Kanazawa University, Kanazawa, Japan; 5 Department of Biochemistry, Tohoku University Graduate School of Medicine, Sendai, Japan; 6 Research Center for Child Mental Development, Kanazawa University, Kanazawa, Japan; 7 Department of Pediatric Otolaryngology, Jichi Children’s Medical Center Tochigi, Jichi Medical University, Tochigi, Japan; 8 Present Address: Department of Diabetes & Cancer Metabolism, City of Hope National Medical Center, Duarte, CA 91010 USA

**Keywords:** Neuroscience, Regeneration and repair in the nervous system

## Abstract

Following facial nerve axotomy, nerve function is not fully restored even after reconstruction. This may be attributed to axon degeneration/neuronal death and sustained neuroinflammation. CD38 is an enzyme that catalyses the hydrolysis of nicotinamide adenine dinucleotide (NAD^+^) and is a candidate molecule for regulating neurodegeneration and neuroinflammation. In this study, we analyzed the effect of CD38 deletion and NAD^+^ supplementation on neuronal death and glial activation in the facial nucleus in the brain stem, and on axon degeneration and immune cell infiltration in the distal portion of the facial nerve after axotomy in mice. Compared with wild-type mice, CD38 knockout (KO) mice showed reduced microglial activation in the facial nucleus, whereas the levels of neuronal death were not significantly different. In contrast, the axon degeneration and demyelination were delayed, and macrophage accumulation was reduced in the facial nerve of CD38 KO mice after axotomy. Supplementation of NAD^+^ with nicotinamide riboside slowed the axon degeneration and demyelination, although it did not alter the level of macrophage infiltration after axotomy. These results suggest that CD38 deletion and supplementation of NAD^+^ may protect transected axon cell-autonomously after facial nerve axotomy.

## Introduction

Facial nerve axotomy is a peripheral motor neuron injury that occurs in trauma and upon surgery. Clinically, the cure rate is low even after nerve reconstruction, and patients often have sustained facial palsy, which leads to aesthetic and psychological consequences^1,2^. Following facial nerve axotomy, both retrograde and anterograde degeneration occur; the former causes cell death in the facial nucleus, and the latter causes Wallerian degeneration^3,4^. During the pathological processes after facial nerve axotomy, the surrounding environment is also dramatically changed, and has an impact on the fate of the transected nerve; astrocytes and microglia are activated in the facial nucleus, and macrophages infiltrate the transected nerve. These cells can produce pro-inflammatory molecules that cause neuropathy, and also enhance the survival of motoneurons, remyelination, and axonal regeneration^3,5,6^. Previous studies using an experimental facial nerve axotomy model revealed that several neurotrophic factors such as ciliary neurotrophic factor (CNTF), brain-derived growth factor (BDNF), and nerve growth factor (NGF) improved neuronal survival or accelerate nerve regeneration^7–9^. There have also been reports for the protective roles of CD4^+^ T cells, anti-inflammatory cytokine IL-10, and gonadal hormones after facial nerve axotomy^10–12^. However, functional recovery was often limited or not significant.

CD38 is a membrane-associated enzyme that converts nicotinamide adenine dinucleotide (NAD^+^) to cyclic adenosine diphosphate-ribose (cADPR)^13–17^. It has been reported that CD38 improves the secretion of insulin from pancreatic beta cells and of oxytocin from hypothalamic neuron, the latter of which has a large impact on the autism spectrum disorder (ASD)-associated social behaviour^16,18–20^. Accumulating evidence suggests that the deletion of CD38 and protection of NAD^+^ reduces neurodegeneration and neuroinflammation^21,22^. We have recently demonstrated that NAD^+^ levels in the brain are higher in CD38 knockout (KO) mice than in wild type (WT) mice, and the demyelination, neuroinflammation, and glial activation are suppressed in CD38 KO mice in cuprizone (CPZ)-induced demyelination model^21^. It was also reported that increased activity of the NAD^+^ biosynthetic enzyme, nicotinamide mononucleotide adenylyl-transferase1 (Nmnat1), was responsible for the axon-sparing activity in Wallerian degeneration slow (wlds) mice^22^. Furthermore, administration of nicotinamide riboside (NR), an NAD^+^ precursor, prevented noise-induced hearing loss and spiral ganglia neurite degeneration^23^. These results suggest that CD38 depletion and high levels of NAD^+^ in the brain suppress neurodegeneration, demyelination, and activation of astrocytes and microglia. However, some studies casted another aspect of NAD^+^-biosynthetic enzymes exerting axon-protective effects, since the protective effects of these enzymes did not correlate with their effect on NAD^+^ levels^24^ and accumulated nicotinamide mononucleotide (NMN)^25^ appeared to be an activator of the prodegenerative cADPR-forming enzyme SARM1^26^. Thus, the neuroprotective effects of NAD^+^ and CD38 on neurodegeneration remain to be clarified.

In this study, we analyzed the effect of CD38 deletion and NAD^+^ repletion on neuronal death and glial activation in the facial nucleus in the brain stem, and on axon degeneration, demyelination and accumulation of immune cells in the distal portion of the facial nerve after axotomy in mice.

## Results

### Histopathological changes in the facial nucleus and facial nerves after facial nerve axotomy

**Figure 1:**
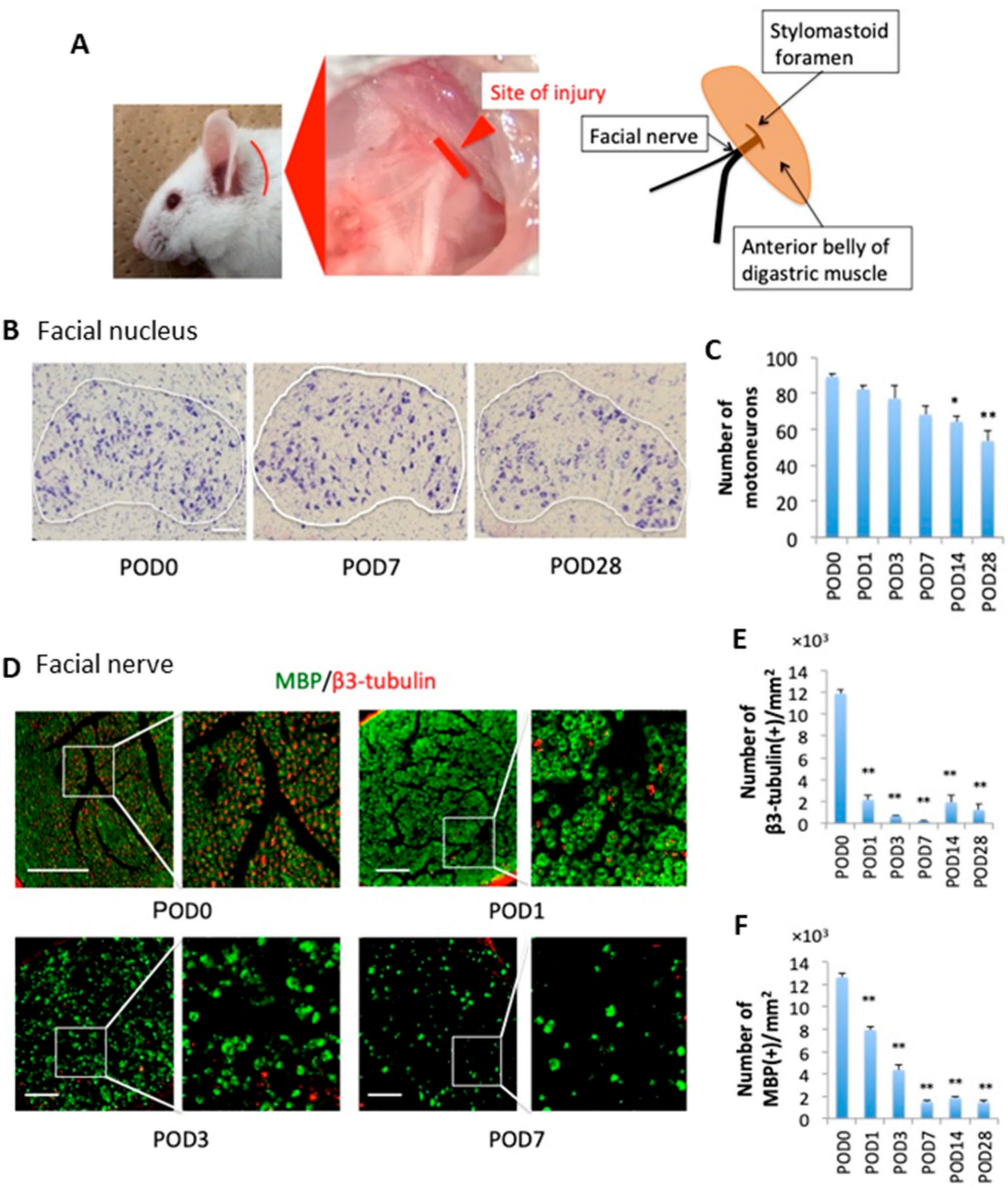
Changes in the facial nucleus and facial nerve structure in WT mice following facial nerve axotomy. (**A**) The facial nerve axotomy in mice. The trunk of the left facial nerve was severed as it exited the stylomastoid foramen. (**B**) Neurons of the facial nuclei stained with Cresyl violet over the course of the experiment. The area circled in white is the facial nucleus. (**C**) The number of surviving cells in the nucleus is shown in relationship to post-operative day (POD) 0, 1, 3, 7, 14, 28, n = 6. (**D**) Cross sections of facial nerves immunostained with myelin basic protein (MBP) (green) and β3-tubulin (red). The images on the right are enlarged views of the areas enclosed in white. (**E**,**F**) The number of axons (**E**) and myelin sheaths (**F**) per 1 mm^2^ of facial nerve sections in relationship to POD. Data are expressed as mean ± SEM. n = 5. *p < 0.05; **p < 0.01 as determined by ANOVA and post-hoc Tukey’s test. Scale bar, 100 μm.

To validate the extent of cell death in the facial nucleus and axon degeneration (Wallerian degeneration) in the distal portion of the facial nerve after axotomy (Fig. 1 A), histological and immunohistochemical analysis were performed using WT (ICR) mice (Fig. 1B–F). Nissl staining revealed that consistent with a previous report^27^, the number of motoneurons in the facial nucleus gradually decreased to ~ 60% of that in the control (sham-operated) mice after facial nerve axotomy (Fig. 1B,C). Immunohistochemistry for myelin basic protein (MBP) and β3-tubulin, which recognize myelin and axonal structures, respectively, revealed that axon degeneration and demyelination started within 1 day, and intact axons and myelin structures were nearly eliminated 7 days after facial nerve axotomy (Fig. 1D–F). Thereafter, the status of axon degeneration and demyelination were investigated until 28 days after axotomy and found no changes (Fig. 1E,F).

### Axon degeneration and demyelination were specifically spared in CD38 KO mice after facial nerve axotomy

**Figure 2:**
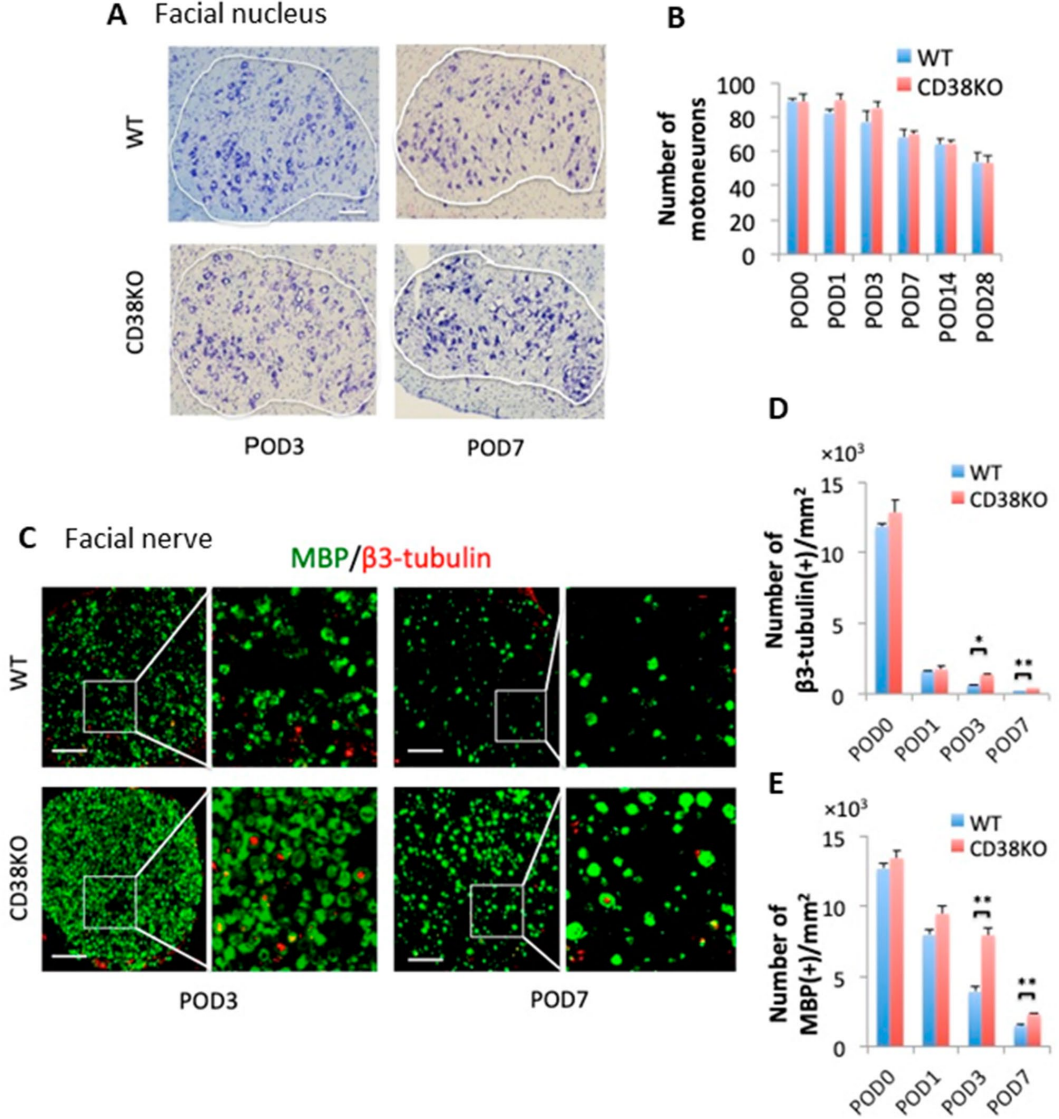
Comparison of facial nerve structures between WT and CD38 knockout (KO) mice following axotomy. (**A**) Nissl staining of neurons in the facial nuclei from WT and CD38 KO mice. The area circled in white is the facial nucleus. (**B**) Time-dependent changes of the number of surviving cells on the nerve transection side (n = 4). (**C**) Cross sections of facial nerves on post-operative days (POD) 3 and POD7 immunostained with myelin basic protein (MBP) (green) and β3-tubulin (red). The images on the right are enlarged views of the areas enclosed in white. (**D**,**E**) The number of axons (**D**) and myelin sheaths (**E**) per 1 mm^2^ of facial nerve sections in relationship to POD. Data are expressed as mean ± SEM. n = 4. *p < 0.05, **p < 0.01, as determined by t-test. Scale bar, 100 μm.

To determine the effect of the deletion of CD38 in our facial nerve axotomy model, the status of neuronal death in the facial nucleus and axon degeneration/demyelination in the facial nerve were analyzed until 28 days after axotomy. Nissl staining revealed that the survival rate of motoneurons in the facial nucleus was not significantly different between WT and CD38 KO mice during the course (Fig. 2A,B). In contrast, immunohistochemistry for MBP and β3-tubulin revealed that axon degeneration and demyelination were both delayed in CD38 KO mice from 3 to 7 days after axotomy (Fig. 2C–E). CD38 KO facial nerve possessed significantly more β3-tubulin-positive and myelinated axons than those in WT mice at POD3 and POD7 (β3-tubulin-positive axons at POD3: *p* = 0.04, POD7: *p* = 0.02. myelinated axons at POD3: *p* = 0.032, POD7: *p* = 0.009) (Fig. 2D,E).

### Microglial activation and infiltration of inflammatory cells were depressed in CD38 KO mice after facial nerve axotomy

**Figure 3:**
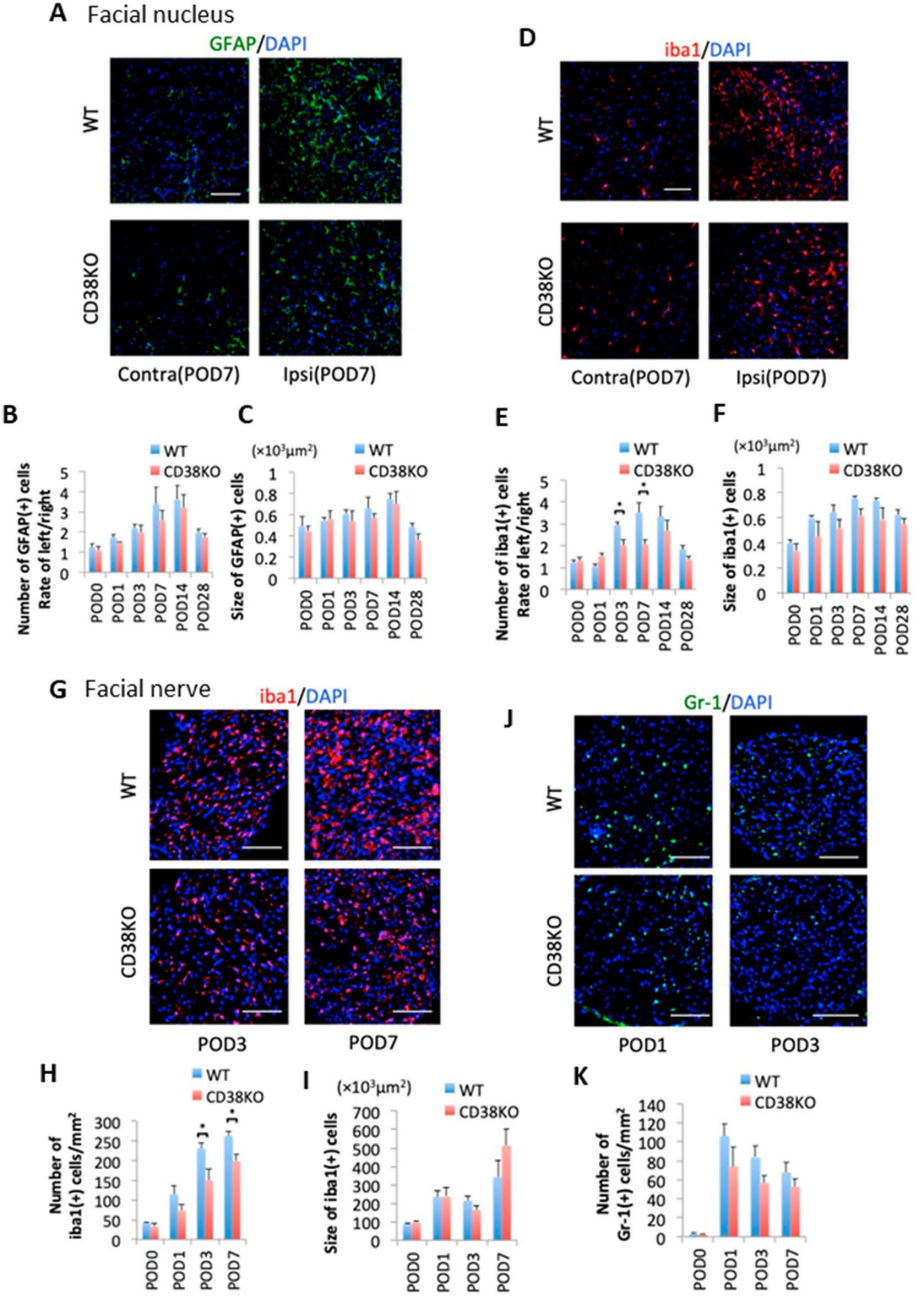
Comparison of time-dependent changes of glial cells and peripheral inflammatory cells between WT and CD38 CD38 knockout (KO) mice. (**A**) Facial nuclei from WT and CD38 KO mice immunostained with glial fibrillary acidic protein (GFAP) (green) and DAPI (blue) at post-operative day (POD) 7. Scale bar, 100 μm. (**B**,**C**) Time-dependent changes in the number (**B**) and the size (**C**) of GFAP + cells. (**D**) Facial nuclei from WT and CD38 KO mice immunostained with Iba1 (red) and DAPI (blue) at POD7. Scale bar, 100 μm. (**E**,**F**) Time-dependent changes in the number (**E**) and the size (**F**) of Iba1 + cells. (**G**,**J**) Cross sections of facial nerves from WT and CD38 KO mice immunostained with Iba1 (**G**) and Gr-1 (**J**) at POD1, POD3, and POD7. Scale bar, 100 μm. (**H**,**I**,**K**) The number (**H**) and the size (**I**) of Iba1-positive cells and the number of Gr-1-positive cells (K). Data are expressed as mean ± SEM. n = 4. *p < 0.05, **p < 0.01, as determined by t-test.

The status of glial activation in the facial nucleus and infiltration of inflammatory cells in the facial nerve was next compared between two genotypes (Fig. 3). Immunohistochemical analyses for glial fibrillary acidic protein (GFAP) and ionized calcium-binding adapter molecule 1 (Iba1), an astroglial and microglial marker, respectively, revealed that the number, but not the size, of microglia was significantly lower in the facial nucleus of CD38 KO mice 3 and 7 days after axotomy (POD3: *p* = 0.038, POD7: *p* = 0.028) (Fig. 3D–F). In contrast, no significant difference was observed either in the number or the size of astrocytes between WT mice and CD38 KO mice, while the number had a tendency to be slightly lower in CD38 KO (Fig. 3A–C).

The infiltration of inflammatory cells was observed in the facial nerve, and the number of Iba1-positive macrophages gradually increased, while the number of Gr-1-positive neutrophils robustly increased at 1 day, then gradually decreased after axotomy. Compared with WT mice, CD38 KO mice had a significantly lower number of Iba1-positive macrophages at POD3 and 7 (POD3: *p* = 0.03, POD7: *p* = 0.02) (Fig. 3G–I). Also, CD38 KO mice had a tendency to have a lower number of Gr-1-positive neutrophils at POD1, 3, and 7; however, the difference did not reach statistical significance (Fig. 3J,K).

### NAD^+^ levels in the facial nuclei and facial nerves were higher in CD38 KO mice

**Figure 4:**
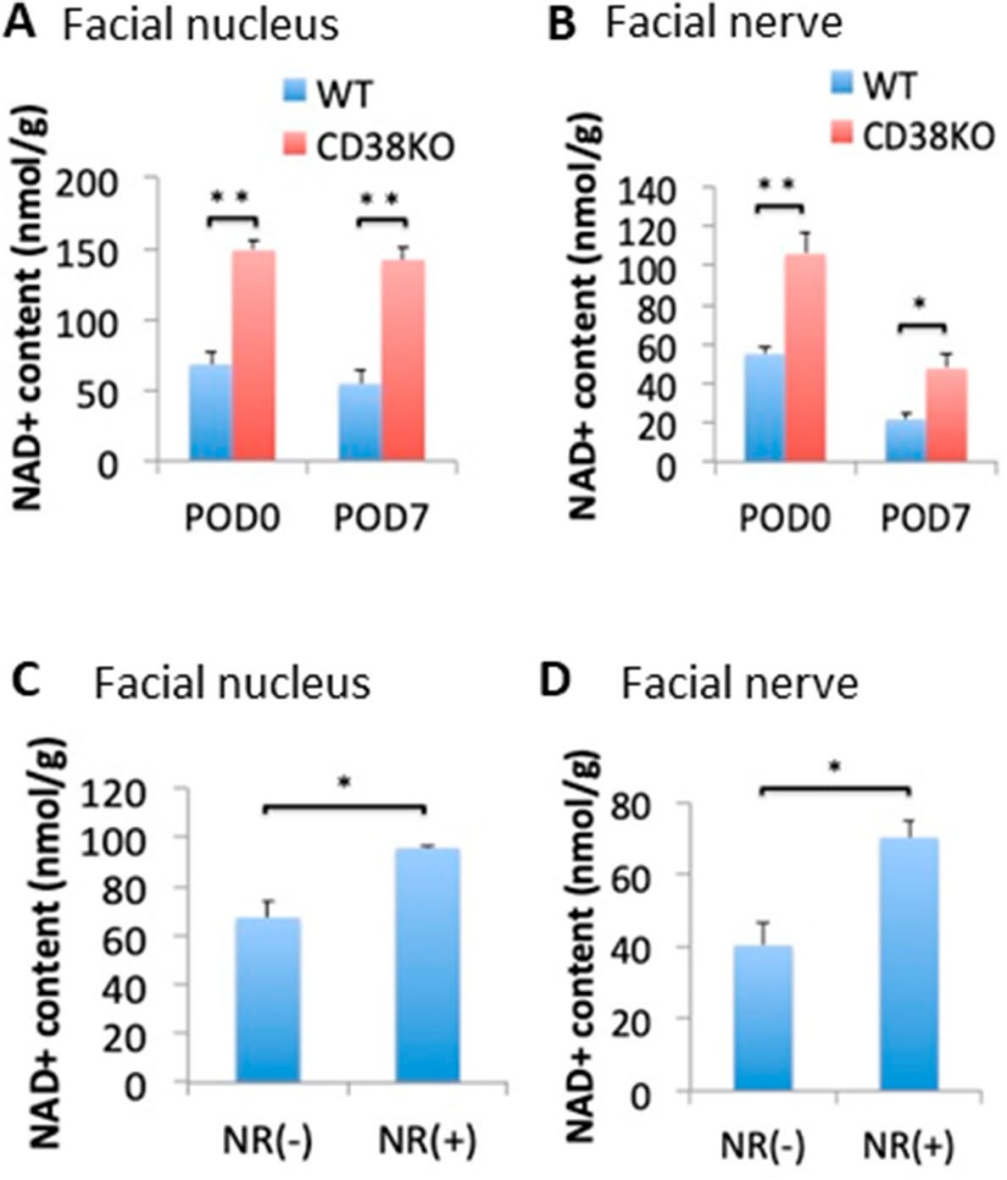
Comparison of the NAD^+^ levels of the facial nuclei and nerves from WT, CD38 KO and nicotinamide riboside (NR)-administered mice. (**A**,**B**) NAD^+^ contents of the facial nuclei (**A**) and nerves (**B**) in WT and CD38 KO mice. (**C**,**D**) NAD^+^ contents of the facial nuclei (**C**) and nerves (**D**) in mice with or without NR administration, NR(+) and NR(−), respectively. Data are expressed as mean ± SEM. n = 4, *p < 0.05, **p < 0.01, as determined by t-test.

To elucidate the possible mechanism of the delayed axon degeneration and demyelination in CD38 KO mice, the NAD^+^ contents were measured in both of the facial nuclei and facial nerves. In the facial nucleus, the NAD^+^ contents did not decrease after axotomy, and the levels were consistently higher in CD38 KO mice (Fig. 4A). In contrast, in the facial nerve, the NAD^+^ contents strongly decreased after axotomy in both genotypes, while the levels were higher in CD38 KO mice (Fig. 4B). These results suggest that deletion of CD38 have an impact on the axon degeneration and demyelination through the higher levels of NAD^+^.

### NR administration to WT mice delayed axon degeneration in facial nerves after axotomy

Nicotinamide riboside (NR) is a form of vitamin B3^28^ that is active in multiple conditions of metabolic stress in which the NAD system is disturbed. These conditions include fatty liver and diabetic neuropathy^29^, heart failure^30^, noise-induced hearing loss, chemotherapeutic neuropathy^31^ and brain injury^32^. We asked whether NAD^+^ boosting with NR had protective effects against axonal degeneration and demyelination in WT mice. In both of the facial nucleus and facial nerve, NAD^+^ levels were significantly higher in NR administered (NR ( +)) WT mice than in control (NR (−)) WT mice (facial nucleus: *p* = 0.016, facial nerve: *p* = 0.01) (Fig. 4C,D), although the levels were lower than those in CD38 KO mice (facial nucleus: *p* = 0.0002, facial nerve: *p* = 0.005) (Fig. 4A,B).

**Figure 5:**
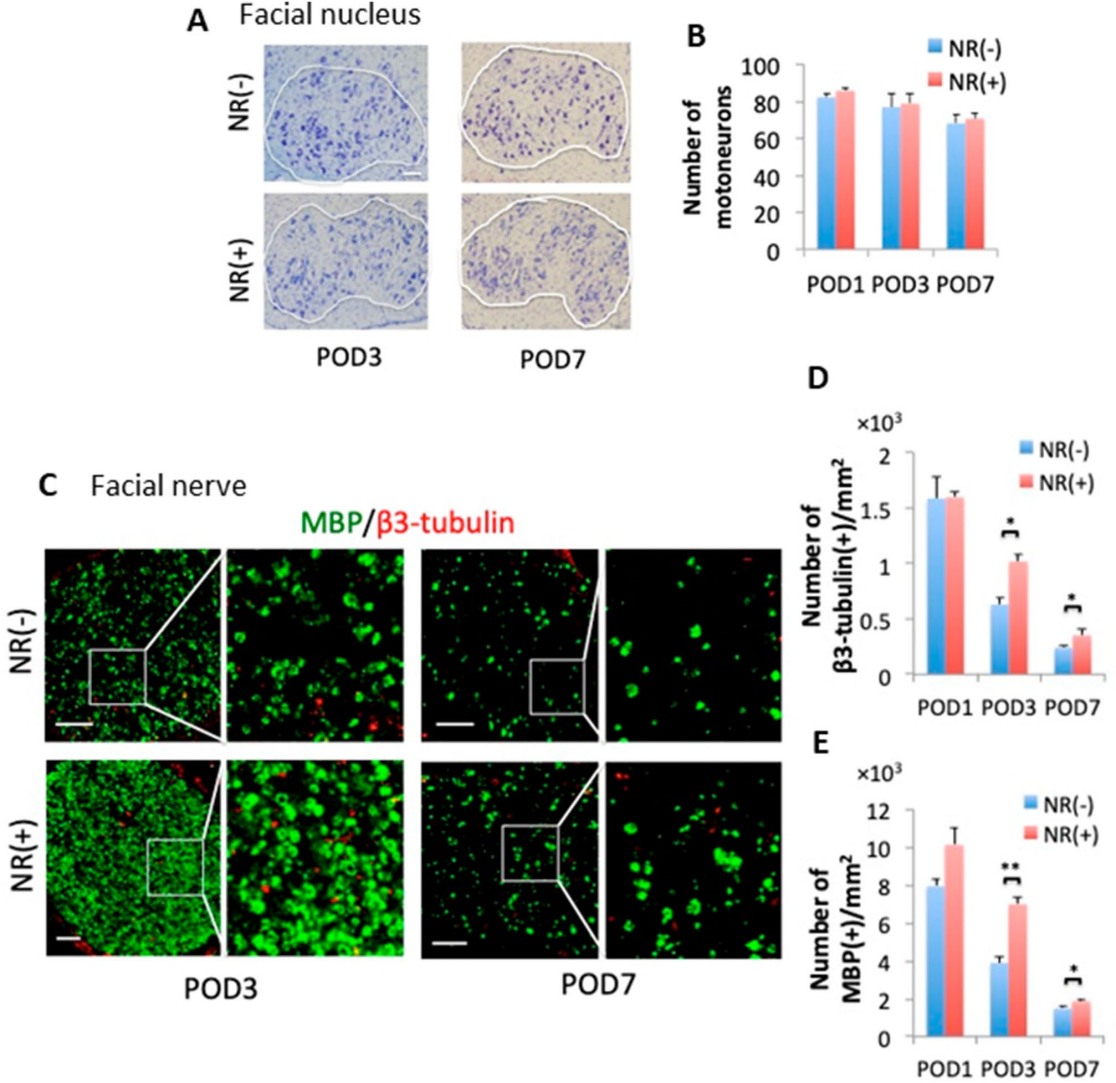
Effects of nicotinamide riboside (NR) administration on the facial nuclei and nerves. (**A**) Nissl staining of neurons in the facial nuclei of WT (NR(−)) and NR-administered WT (NR( +)) mice. The area circled in white is the facial nucleus. (**B**) The time-dependent changes of the number of surviving cells on the nerve transection side in NR− and NR + mice (n = 4). (**C**) Facial nerve cross-sections from NR − and NR + mice at POD3 and POD7 immunostained with myelin basic protein (MBP) (green) and β3-tubulin (red). The images on the right are enlarged views of the areas enclosed in white. (**D**,**E**) Time-dependent changes in the number of axons (**D**) and myelin sheaths (**E**) per mm^2^ in facial nerve sections. Data are expressed as mean ± SEM. n = 4. *p < 0.05, **p < 0.01, as determined by t-test. Scale bar, 100 μm.

Nissl staining revealed that the survival rate of motoneurons in the facial nucleus was not significantly different between NR (+) and NR (−) mice after axotomy (Fig. 5A,B). In contrast, immunohistochemistry for MBP and β3-tubulin revealed that axon degeneration and demyelination were delayed in NR (+) mice from 3 to 7 days after axotomy (Fig. 5C–E).

**Figure 6:**
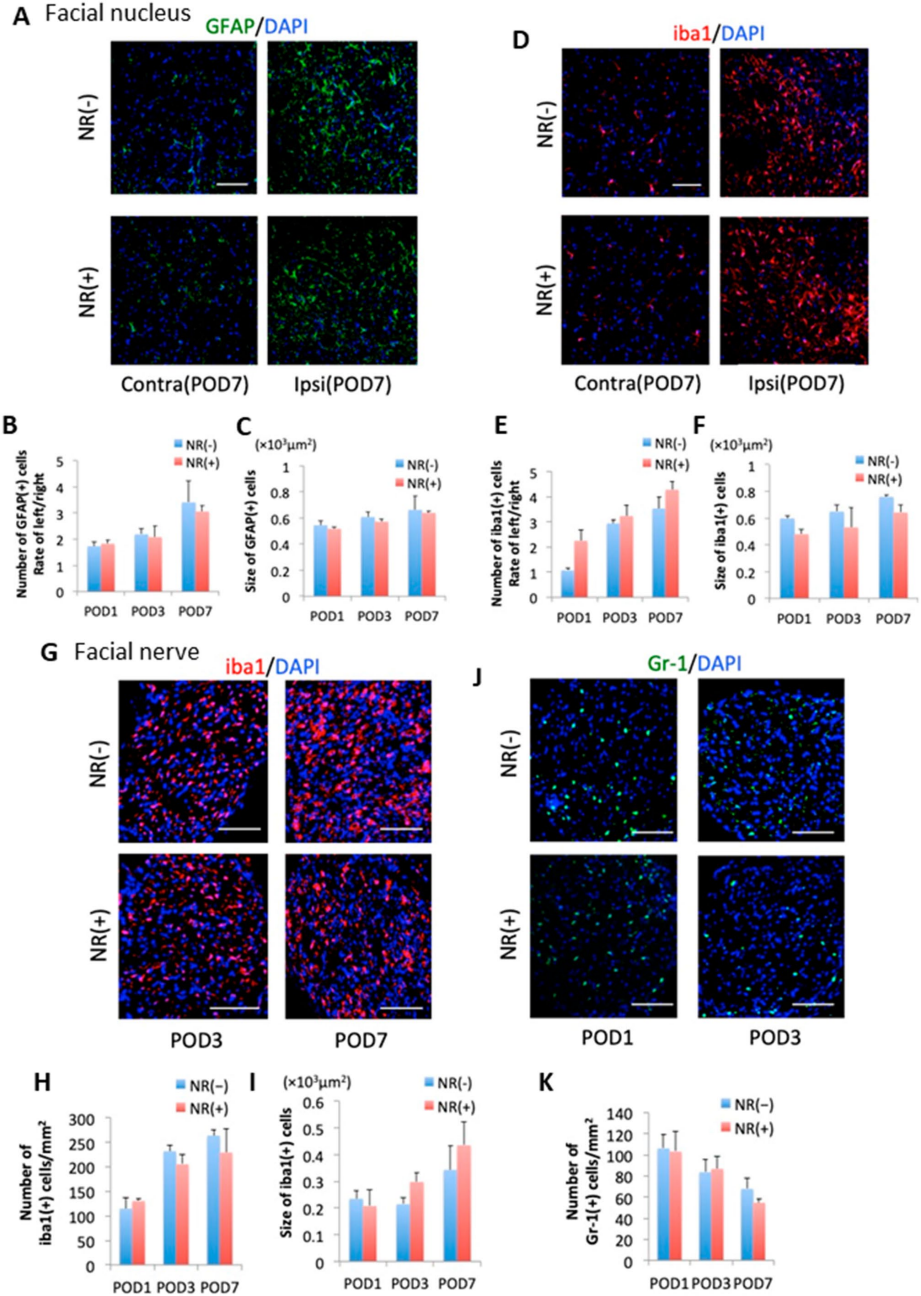
Comparison of time-dependent changes of glial cells and peripheral inflammatory cells in WT mice with and without nicotinamide riboside (NR) administration. (**A**) Facial nuclei from WT (NR(−)) and NR-administered WT (NR( +)) mice immunostained with GFAP (green) and DAPI (blue) at POD7. Scale bar, 100 μm. (**B**,**C**) Time-dependent changes in the number (**B**) and the size (**C**) of GFAP + cells. (**D**) Facial nuclei of WT and NR mice immunostained with Iba1 (red) and DAPI (blue) at POD7. Scale bar, 100 μm. (**E,F**) Time-dependent changes in the number (**E**) and the size (**F**) of Iba1 + cells. (**G**,**J**) Cross-sections of facial nerves from WT and NR mice immunostained with Iba1 (**G**) and Gr-1 (**J**) at POD1, POD3, and POD7. (**H**–**K**) The numbers (**H**) and the size (**I**) of Iba1-positive cells and the number of Gr-1-positive cells (**K**). Data are expressed as mean ± SEM. n = 4, respectively. *p < 0.05, **p < 0.01, as determined by t-test.

Immunohistochemical analysis also revealed that the number and the size of GFAP-positive astrocytes and Iba1-positive microglia were at similar levels in the facial nucleus between NR (+) and NR (−) mice after axotomy (Fig. 6A–F). Similarly, the number of Iba1-positive macrophages and Gr-1-positive neutrophils were at similar levels in the facial nerves of NR (+) and NR (−) mice after axotomy (Fig. 6G–K). Thus, a higher NAD^+^ status was linked to greater resistance to axonopathy in WT mice without an apparent improvement in neuroinflammatory parameters.

## Discussion

In the current study, we investigated the effect of CD38 deletion and NR administration in a mouse facial nerve axotomy model. Deletion of CD38 delayed axon degeneration and demyelination (Fig. 2C–E) and suppressed infiltration of macrophage at the distal portion of the transected nerve (Fig. 3H). In contrast, neuronal survival in the facial nucleus was not significantly different between two genotypes (Fig. 2A,B), even though the accumulation of microglia was suppressed in CD38 KO mice (Fig. 3E). Although previous reports demonstrated that the deletion of CD38 reduced neurodegeneration and suppressed glial activation in the central nervous system^33–36^, our results suggest that CD38 deletion particularly affects the axon degeneration, demyelination, and macrophage inflammation at the local site of the peripheral nerve. It is not yet clear why neuronal survival in the facial nucleus was unchanged in CD38 KO mice after axotomy, although NAD^+^ level was higher and the accumulation of microglia was lower than those in WT mice. We, however, speculate that activated microglia have both beneficial and detrimental effects on the neuronal survival after axotomy, as previously reported^37,38^ and both effects are suppressed by deleting CD38. In addition, our model causes relatively mild damage to the neuronal cells in the facial nucleus compared to the intracranial axotomy model, in which the facial nerve is transected at the portion closer to the brain stem^39^, such that the rescuing effects may be difficult to observe.

In facial nerves, our results suggest that the increase of NAD^+^ is more directly associated with preservation of the axon than suppression of immune cell infiltration. However, it is important to consider that multiple NAD^+^ metabolites and targets of NAD^+^ metabolism play critical roles in neurodegeneration in multiple cell types. Peripheral nerves have served as an important model that may enlighten our study of facial nerves. Because peripheral nerve axons are so elongated, they are highly dependent on fast axonal transport and NAD^+^-dependent bioenergetics along the length of the axon. NAD^+^ synthesis in axons depends on the Golgi-associated NMN adenyltransferase 2 (NMNAT2), an unstable protein that converts cytosolic NMN to NAD^+^^40^. Upon nerve damage, NMN levels rise, which leads to activation of SARM1^23^, an enzyme required for degeneration^41^, that converts NAD^+^ to cADPR^42^. In multiple models of neurodegeneration, genes encoding NR kinases are transcriptionally elevated^32,43^ and NR has been shown to be neuroprotective in vitro^43^ and in vivo^29,31^. The transcriptional induction of the NR kinase pathway is rationalized by the lower ATP cost to produce NAD^+^ from NR when a tissue is in a bioenergetic crisis^30^. Besides the effect on axon degeneration, deletion of CD38 and administration of NR may have effects on Schwann cells, also knowns as key players in myelin clearance^44^. These points should be clarified in the future studies.

Functional neuroprotection by NR reminded us with wlds mice, in which Wallerian degeneration was found to be about tenfold slower than in WT animals, and macrophage infiltration was decreased^45^. Importantly, the delay in Wallerian degeneration was not caused by mutation of the NMNAT gene in macrophages but presumably in the peripheral neurons. In wlds mice, the nuclear NMN adenylyltransferase 1 (NMNAT1) was mislocalized into the axonoplasm where the NAD^+^ level was maintained and accumulation of NMN was prevented cell-autonomously^46^. However, damaged neurons were likely to reach a point of no return if they no longer had NMNAT2 activity in the axonoplasm, and, in fact, the combination of nicotinic acid riboside (NAR) plus FK866 was shown to be more neuroprotective than NR in an ex vivo model of neuroprotection by virtue of protecting NAD^+^ without formation of NMN^47^.

In WT mice that are provided with NR, protection against axonopathy would likely depend on transcriptional induction of NR kinase genes^32,43^ and presence of residual NMNAT2 in order to maintain NAD^+^ and prevent neurotoxic accumulation of NMN^26^. While deletion of CD38 from neurons would be expected to protect against loss of NAD^+^ that would follow loss of NMNAT2 activity, the effects seem to be quite smaller than those in wlds mice. Furthermore, it is difficult to see how CD38 deletion has axon-sparing activity in a cell autonomous manner if SARM1 activation and its intracellular production of cADPR are necessary and sufficient to drive Wallerian activity. We are therefore planning to test whether CD38 KO derived neurons or CD38-inhibited WT neurons are protected against axonopathy; if they are, then it suggests that either SARM1 is inhibited by higher levels of NAD or that CD38-dependent cADPR production may contribute to calcium and calpain-induced axonopathy^48^ in a manner parallel to that initiated by SARM1. Such an activity would presumably be mediated by the type III membrane orientation of CD38 with the active site in the cytosol^49^. It is crucial to remember, however, that CD38 is not only deleted from neurons in our model but throughout the body. Currently, we cannot exclude the possibility that CD38-related secretory molecules such as insulin^16^ and oxytocin^18^ play indirect, but important roles in Wallerian degenerationafter axotomy, as previously described^50,51^. Deletion of CD38 from astrocytes and microglia would also be expected to reduce inflammatory gene expression^21^. Elevated NAD^+^ status conferred by three weeks of oral NR was similarly shown to lower circulation of inflammatory markers in older humans^52^. Thus, through the use of cell type-specific knockouts and pharmacological interventions, we would determine the specific mechanisms of NR and CD38-mediated neuroprotection in order to help improve human health.

In the clinical field, the most common surgical approach for repairing transected nerves has been direct suture of the two stumps (end-to-end anastomosis) but functional recovery is poor^49^. Also, when the nerve gap is larger than 5-mm, nerve interposition or “cable graft” procedures are commonly performed. However, the recovery of the transected or resected nerve after reconstruction of the nerve defect is not satisfactory^53^. Thus, the treatment of functional recovery after facial nerve axotomy is still a major problem. Our result may raise the possibility of the treatment for facial nerve axotomy from the view of suppressing nerve degeneration by focusing on NAD^+^ metabolism. Combining NAD^+^ supplementation/CD38 inhibition and surgical treatment may become the new treatment to improve the outcome of facial nerve axotomy.

In conclusion, we have shown that, following facial nerve axotomy, axon degeneration, demyelination, and infiltration of immune cells are suppressed in CD38 KO mice compared with that in WT mice. Furthermore, supplementation of NAD^+^ with NR slowed the axon degeneration and demyelination in WT mice. Further study with different compounds modulating NAD^+^ metabolism may prove new candidates for the therapeutic targets in the facial nerve injury.

## Materials and methods

### Animals

WT and CD38 KO male mice in the ICR background were used for the experiments (body weight: 35–40 g). CD38 KO mice were generated at Tohoku University as previously described^14^, and backcrossed with ICR mice (SANKYO LABO SERVICE CORPORATION, INC., Tokyo, Japan) at least eight times at Kanazawa University. All mice were housed in mouse cages (345 mm × 168 mm × 140 mm) in a temperature-controlled room (24 °C) with a 12:12 light: dark cycle. The animals were sacrificed at post-operative day (POD) 1, 3, 7, 14, and 28. All animal experiments were performed in accordance with the guidelines of and approved by the Animal Care and Use Committee of Kanazawa University (AP-183960).

### NR administration model

Mice were administered NR (NIAGEN, ChromaDex, CA, USA) (400 mg/kg) via intraperitoneal injection once daily for one week prior to facial nerve axotomy, as previously described^22^.

### Surgical procedure

For systemic anaesthesia, a combination anaesthetic including 0.3 mg/kg of medetomidine (Orion Corporation, Espoo, Finland), 4.0 mg/kg of midazolam (Maruishi Pharmaceutical Co., Ltd., Osaka, Japan), and 5.0 mg/kg of butorphanol (Meiji Seika Pharma Co., Ltd., Tokyo, Japan), was injected intraperitoneally. After anaesthesia, a postauricular incision was made under a microscope to reveal the peripheral facial nerve at the stylomastoid foramen. The nerve was severed with spring scissors the severed ends were pushed away from each other to avoid reconnection (Fig. 1A).

### Histopathology

After perfusion with 4% paraformaldehyde (PFA), the brain stem and bilateral facial nerve were removed from mice post fixated in 4% PFA, followed by dehydration in 30% sucrose. Coronal sections were cut at a thickness of 17 μm with a cryostat (CM1950, Leica, Nussloch, Germany). To evaluate motoneurons in the facial nucleus, the sections were processed for histopathological staining with cresyl violet (Nissl staining). The image was analysed by light microscope (IX83P2-CAS-D8-SP, Olympus, Tokyo, Japan) at 10 × magnification, and the number of motoneurons in the facial nucleus was measured using 4 sections per mouse.

### Immunohistochemistry

To evaluate axonal degeneration and demyelination, sections were processed for immunostaining with antibodies against MBP (1:400 Merck Millipore, Burlington, MA, USA) and β3-tubulin (1:400 Sigma-Aldrich, St. Louis, MO, USA). For the detection of GFAP and Iba1, in the facial nucleus, sections were processed for immunostaining with antibodies against GFAP (1:400, Sigma-Aldrich, St. Louis, MO, USA), Iba1 (1:400, Wako Pure Chemical Industries, Osaka, Japan)^54^, For the evaluation of inflammatory cells in the facial nerve, the sections were processed for immunostaining with antibodies against Iba1 (1:300) to identify macrophages^55^ and Gr-1 (1:200, Abcam, Cambridge, UK) to identify neutrophils^56^. Alexa 488-conjugated secondary antibodies (1:200, Abcam) or Cy3-conjugated secondary antibodies (1;100, Abcam) were used to visualize immunolabeling. Immunofluorescent staining of primary cultures was performed as previously described^36^. Imaging was performed by a laser scanning confocal microscope (Eclipse TE200U, Nikon, Tokyo, Japan) with Nikon EZ-C1 software or by a fluorescence microscope (BZ-X710, Keyence, Osaka, Japan) at 20 × magnification. The number of GFAP-, Iba1-, and Gr-1-positive cells with 4′,6-diamidino-2-phenylindole (DAPI)-positive nuclei in the facial nucleus was measured using 4 sections per mouse. The results were presented as the number of axons and myelin sheaths per mm^2^ for axonal degeneration, demyelination, respectively, the ratio of the number of inflammatory cells in left facial nuclei against the right ones, and the number of inflammatory cells in facial nerves per mm^2^.

### Analysis of NAD^+^ levels in the facial nucleus and facial nerve

Mice were anesthetized with a combination anaesthetic (0.3 mg/kg of medetomidine, 4.0 mg/kg of midazolam, and 5.0 mg/kg of butorphanol) injected intraperitoneally, and then the facial nucleus and facial nerve were removed. For analysis of NAD^+^ levels, we used an EnzyChrom NAD^+^/NADH Assay Kit (E2ND-100, BioAssay Systems, Hayward, CA, USA). The brain stems and facial nerves of WT, CD38 KO, and NR-administered mice were harvested, weighed, and homogenized in NAD extraction buffer. The extracts were heated at 60 °C for 5 min and then the Assay Buffer and NADH extraction buffer were added to neutralize the extract. The sample was briefly vortexed and spun down for 5 min. The supernatant was then used for the NAD assays. These samples were incubated in the Working Reagent (a mix of the Assay buffer, Enzyme A, Enzyme B, Lactate, and MTT Solution) and the optical density was immediately measured at 565 nm and then again after a 15 min incubation period at room temperature (20 °C). Standard Curve was made by using NAD Premix solution in distilled water in the kit.

### Statistics

Analysis of variance (ANOVA) was performed for statistical comparisons involving the number of motoneurons, astrocytes, and microglia in the facial nucleus; as well as the axons, myelin sheaths, and inflammatory cells at different development stages. The *p* value for post-hoc pairwise comparisons were adjusted using the Tukey method. A Student’s t-test was employed between WT and CD38 KO mice, as well as between mice with and without NR administration. Statistical significance was defined as *p* < 0.05.
